# Exploring the hybrid speciation continuum in birds

**DOI:** 10.1002/ece3.4558

**Published:** 2018-12-05

**Authors:** Jente Ottenburghs

**Affiliations:** ^1^ Resource Ecology Group Wageningen University Wageningen The Netherlands

**Keywords:** adaptation, admixture, genomics, introgression, mito‐nuclear discordance, reproductive isolation

## Abstract

Hybridization is increasingly recognized as a creative evolutionary force contributing to adaptation and speciation. Homoploid hybrid speciation—the process in which hybridization results in a stable, fertile, and reproductively isolated hybrid lineage where there is no change in ploidy—has been documented in several taxa. Hybridization can directly contribute to reproductive isolation or reinforce it at a later stage. Alternatively, hybridization might not be related to the evolution of reproductive isolation. To account for these different scenarios, I propose to discriminate between two types of hybrid speciation: type I where reproductive isolation is a direct consequence of hybridization and type II where it is the by‐product of other processes. I illustrate the applicability of this classification scheme with avian examples. To my knowledge, seven hybrid bird species have been proposed: Italian sparrow, Audubon's warbler, Genovesa mockingbird, Hawaiian duck, red‐breasted goose, golden‐crowned manakin, and a recent lineage of Darwin's finches on the island of Daphne Major (“Big Bird”). All studies provide convincing evidence for hybridization, but do not always confidently discriminate between scenarios of hybrid speciation and recurrent introgressive hybridization. The build‐up of reproductive isolation between the hybrid species and their parental taxa is mainly driven by premating isolation mechanisms and comparable to classical speciation events. One hybrid species can be classified as type I (“Big Bird”) while three species constitute type II hybrid species (Italian sparrow, Audubon's warbler, and golden‐crowned manakin). The diversity in hybrid bird species across a range of divergence times also provides an excellent opportunity to study the evolution of hybrid genomes in terms of genome stabilization and adaptation.

## 1 INTRODUCTION

The Greek philosopher Aristotle extensively described hybrids in his *Historia Animalium* and even called upon hybridization to explain the species richness in Africa: “And the proverb about Libya [Africa], that ‘Libya is always producing something new,’ is said to have originated from animals of different species uniting with one another in that country, for it is said that because of the want of water all must meet at the few places where springs are to be found, and that even different kinds unite in consequence.” Similarly, Linnaeus considered hybridization as a creative force in the origin of new species (Linnaeus, 1751). In *Disquisitio de Sexu Plantarum,* he stated that “it is impossible to doubt that there are new species produced by hybrid generation.” This idea, hybrid speciation, is now defined as “the process in which natural hybridization results in the production of an evolutionary lineage that is at least partially reproductively isolated from both parental lineages and demonstrates a distinct ecological trajectory” (Arnold, 2006).

Two principal categories of hybrid speciation are recognized, homoploid and alloploid hybrid speciation, based on whether or not the ploidy level changes (Mallet, 2007). In this paper, I will focus on the first category, homoploid hybrid speciation (hereafter HHS), which results in a stable, fertile, and reproductively isolated hybrid lineage in which there is no change in ploidy level (Mallet, 2007; Mavarez & Linares, 2008). Schumer, Rosenthal, and Andolfatto (2014) argued that three criteria should be satisfied in order to indisputably demonstrate HHS: (a) genetic or morphological evidence for hybridization, (b) reproductive isolation of the hybrid lineage from its parental species, and (c) evidence that reproductive isolation is a direct consequence of past hybridization. Of the many putative cases of HHS among plants (Gross & Rieseberg, 2005; Rieseberg, 1997) and the few among animals (Mavarez & Linares, 2008), only three plant species (Rieseberg et al., 2003) and one species of butterfly (Mavarez et al., 2006) meet all three criteria.

Some authors have argued that criterion three is too stringent (Feliner et al., 2017). For example, this criterion cannot be satisfied when a hybrid population is geographically isolated from its parental species for a certain amount of time, during which reproductive isolation may develop as a result of random drift or divergence (Brelsford, 2011; Hermansen et al., 2011). In this case, reproductive isolation is not the direct result of hybridization, but the by‐product of a geographical isolation phase in the hybrid speciation process. To account for such scenarios, I propose to differentiate between two types of hybrid speciation: type I where reproductive isolation is a direct consequence of hybridization and type II where reproductive isolation is the by‐product of other processes, such as geographical isolation. One could define subtypes within type II hybrid speciation, such as “allopatric” or “ecological” scenarios, but this might quickly lead to a “balkanization” of speciation scenarios. The current proposal only refers to the relationship between hybridization and reproductive isolation at the onset of hybrid speciation.

Apart from the epistemological discussion on the definition of a hybrid species (Feliner et al., 2017; Schumer, Rosenthal, & Andolfatto, 2018), it is important to understand the process of hybrid speciation itself. Indeed, Schumer, Rosenthal, et al. (2018) noted that “observing hybrid ancestry in the genome provides direct evidence that the species has an admixed genome (or even a genome of hybrid origin) but does not necessarily tell us about the evolutionary processes that give rise to that species.” The rapid progress in genomic sequencing techniques has revealed several putative hybrid species (Mallet, 2007; Mavarez & Linares, 2008; Schumer et al., 2014), providing an excellent opportunity to study the origin of these hybrid lineages.

Similar to classical speciation scenarios, one can envision several stages along the hybrid speciation continuum (Seehausen et al., 2014). The main difference between classical speciation scenarios and hybrid speciation events is the initial phase of admixture (Nolte & Tautz, 2010). Hybridization between two lineages can result in the formation of a hybrid swarm of which the members are not reproductively isolated from the parental taxa. Over time, reproductive isolation mechanisms might develop and the hybrid swarm starts to follow its own distinct evolutionary trajectory. Different hybrid lineages will occupy different positions along this continuum, ranging from hybrid swarms over partially reproductively isolated lineages to independently evolving hybrid species. Studying hybrid lineages along the speciation continuum can provide important insights into several evolutionary processes, such as the role of hybridization in the build‐up of reproductive isolation and the consequent evolution of hybrid genomes (Comeault, 2018; Schumer, Xu, et al., 2018).

## 2 PUTATIVE HYBRID BIRD SPECIES

Here, I will illustrate the applicability of the type I and type II classification scheme for hybrid species by exploring the hybrid speciation continuum in birds, which are prone to hybridize (Ottenburghs, Ydenberg, van Hooft, van Wieren, & Prins, 2015). To my knowledge, seven bird species have been proposed to have hybrid origins: Italian sparrow (*Passer italiae*), Audubon's warbler (*Setophaga auduboni*), Genovesa mockingbird (*Mimus parvulus bauri*), Hawaiian duck (*Anas wyvilliana*), red‐breasted goose (*Branta ruficollis*), golden‐crowned manakin (*Lepidothrix vilasboasi*), and a recent lineage of Darwin's finches on the island of Daphne Major (originally referred to as “Big Bird”). In the following paragraphs, I discuss the evidence supporting the hybrid origin of these species.

### 2.1 Italian Sparrow

Captive‐bred hybrids between house sparrow (*Passer domesticus*) and Spanish sparrow (*P. hispaniolensis*) so resemble the Italian sparrow (*P. italiae*) that it was hypothesized to be of hybrid origin (Töpfer, 2006). The Italian Sparrow shares mitochondrial haplotypes with both parental species and genetic analyses of both microsatellite data and nuclear sequences already indicated an admixed nuclear genome (Elgvin et al., 2011; Hermansen et al., 2011). These results were later confirmed by genomic data (Elgvin et al., 2017; Runemark, Trier, et al., 2018). The hybrid speciation event probably occurred less than 10,000 years ago when house sparrows expanded across Europe and came into contact with the Spanish sparrow (Hermansen et al., 2011).

The Italian Sparrow appears to be reproductively isolated from the Spanish sparrow, because no signs of interbreeding were detected in a sympatric area on the Gargano Peninsula in Italy (Hermansen et al., 2011). In contrast, the Italian sparrow does interbreed with the house sparrow in the Alps (Lockley, 1992, 1996 ), but mito‐nuclear and sex‐linked incompatibilities probably result in partial reproductive isolation between these species (Hermansen et al., 2014; Trier, Hermansen, Sætre, & Bailey, 2014).

### 2.2 Audubon's Warbler

The yellow‐rumped warbler (*Setophaga coronata*) complex comprises four distinct taxa—*coronata*,* auduboni*,* nigrifrons* and *goldmani*—of which the taxonomic relationships remain controversial (Hubbard, 1969; Mila, Smith, & Wayne, 2006). A survey of mitochondrial and nuclear genetic variation supports the idea that Audubon's warbler (*S. auduboni*) is a hybrid lineage between myrtle warbler (*S. coronata*) and black‐fronted warbler (*S. nigrifrons*) (Brelsford, Mila, & Irwin, 2011). Audubon's warbler is partially reproductively isolated from myrtle warbler by postmating barriers (Brelsford & Irwin, 2009) and a migratory divide between Audubon's warbler and myrtle warbler could contribute to reproductive isolation if hybrids show suboptimal migration strategies (Toews, Brelsford, & Irwin, 2014; Toews, Mandic, Richards, & Irwin, 2014).

Reproductive isolation between Audubon's warbler and black‐fronted warbler has not yet been assessed, although there appears to be a cryptic hybrid zone in southern Utah (Mila, Toews, Smith, & Wayne, 2011). Further studies are warranted to quantify the degree of reproductive isolation between Audubon's Warbler and its parental species. The hybrid speciation event probably involved an allopatric phase; the authors suggest that “hybridization in a previous interglacial period followed by persistence in a glacial refugium and subsequent expansion across western North America may be the most straightforward explanation for the existence of [Audubon's warbler] as a distinct form with a recognizable phenotype and genotype over a broad region” (Brelsford et al., 2011). Hence, Audubon's warbler is probably a type II hybrid species.

### 2.3 Genovesa Mockingbird

On the Galapagos Archipelago, the Genovesa Mockingbird (*Mimus parvulus bauri*) shares mitochondrial haplotypes and certain autosomal loci with the San Cristobal Mockingbird (*M. melanotis*). At other autosomal loci, it clusters closely with another species, the Galapagos Mockingbird (*M. parvulus*). These observations suggest that the Genovesa Mockingbird represents a lineage of hybrid ancestry (Nietlisbach et al., 2013). However, the pattern of genetic mosaicism of the Genovesa Mockingbird can also be the result of repeated events of introgressive hybridization. Furthermore, the strength of reproductive isolation between this taxon and its putative parental species remains unknown. Although Nietlisbach et al. (2013) argue that “this case could be considered one of incipient homoploid hybrid speciation”, more research is necessary to support this claim.

### 2.4 Hawaiian Duck

The Hawaiian Duck is one of the 14 taxa within the mallard (*Anas platyrhynchos*) complex, a phylogenetically challenging group of ducks (Lavretsky, McCracken, & Peters, 2014). The relationships within this complex remain uncertain, including the position of the Hawaiian duck. Morphological data (Livezey, 1991) and nuclear DNA (Lavretsky et al., 2014) support a sister relation with the Laysan duck (*Anas laysanensis*), whereas mtDNA clusters the Hawaiian duck with the mallard. This pattern of morphological‐nuclear‐mitochondrial discordance can be explained by incomplete lineage sorting or introgressive hybridization.

Several lines of evidence suggest this discordance is not only the outcome of introgression, but that the Hawaiian duck might even be a young hybrid species (Lavretsky, Engilis, Eadie, & Peters, 2015). Gene flow from both parental species is necessary to explain the genetic diversity in the Hawaiian duck. Only one SNP is fixed within Hawaiian duck, the remaining SNPs were intermediate between the parental species. This genetic mosaicism was further supported by Bayesian clustering and isolation‐with‐migration analyses. Moreover, fossil remains point to a hybrid origin: The Hawaiian fossil record contains Laysan‐like ducks from the mid‐Pleistocene and intermediate Laysan‐Hawaiian ducks from the Holocene (Cooper et al., 1996; Olson & James, 1991). Together these observations point to a hybrid speciation event about 3,000 years ago. Currently, Hawaiian ducks still interbreed with mallards. The hybrid offspring are viable and fertile, indicating that reproductive isolation is mainly driven by premating mechanisms (Fowler, Eadie, & Engilis, 2009).

### 2.5 Red‐breasted Goose

The seventeen species of True Geese are classified in the waterfowl tribe Anserini and have been traditionally divided over two genera: *Anser* and *Branta* (Ottenburghs, Megens, et al., 2016). A phylogenomic study of this tribe suggested that the red‐breasted goose might be of hybrid origin (Ottenburghs, Megens, et al., 2017). Phylogenetic networks and d‐statistics indicated ancient admixture between brent goose (*Branta bernicla*) and the ancestor of the white‐cheeked geese (i.e., Hawaiian goose [*B. sandvicensis*], Canada goose [*B. canadensis*], cackling goose [*B. hutchinsii*], and barnacle goose [*B. leucopsis*]). The putative hybrid speciation event happened at least 3.5 million years ago, before the diversification of the white‐cheeked geese clade. The red‐breasted goose seems partially reproductively isolated from its parental species; some hybrids have been documented, but their fertility has not been directly assessed (Ottenburghs, van Hooft, van Wieren, Ydenberg, & Prins, 2016).

### 2.6 Golden‐crowned Manakin

In Amazonian Brazil, the geographic range of golden‐crowned manakin (*Lepidothrix vilasboasi*) is located between the ranges of opal‐crowned (*Lepidothrix iris*) and snow‐capped manakin (*L. nattereri*). Genetic analyses suggest that the golden‐crowned manakin is the outcome of a hybrid speciation event between the latter two species about 158,000 years ago (Barrera‐Guzmán, Aleixo, Shawkey, & Weir, 2018a). The golden‐crowned manakin derived about 15%–20% of its genome from snow‐capped manakin and the remainder from opal‐crowned manakin. The authors suggest that the initial hybridization events were followed by periods of allopatry due to the combined effects of river barriers and forest contraction during past climatic oscillations. If this scenario is correct, the golden‐crowned manakin would constitute a type II hybrid species.

It is not known whether the golden‐crowned manakin has developed complete reproductive isolation from its parents. There are probably parapatric contact zones in the western and southern parts of its range that allow for assessing the strength of reproductive isolation. However, some premating isolation might be based on crown‐color signals. The parental species have highly reflective crown patches, whereas the golden‐crowned manakin has less reflective yellow plumage. To compensate for the reduced brightness of the crown patch in the hybrid species, sexual selection might have resulted in thickening of the crown patch with carotenoids, culminating in the yellow color (Barrera‐Guzmán et al., 2018a). Whether the yellow crown patch directly led to reproductive isolation or whether it reinforces reproductive isolation in combination with other pre‐ and postzygotic isolation mechanisms remains to be investigated (Barrera‐Guzmán, Aleixo, Shawkey, & Weir, 2018b; Rosenthal, Schumer, & Andolfatto, 2018).

### 2.7 “Big Bird”

The intensive study of Darwin's Finches on the small Galapagos Island of Daphne Major (Grant & Grant, 2014) might have documented a very recent case of HHS. In 1981, a large cactus finch (*Geospiza conirostris*) arrived on the island and mated with a female medium ground finch (*G. fortis*). The resulting offspring only bred with each other and as such established a new lineage on the island (Grant & Grant, 2009; Lamichhaney et al., 2018). This lineage is reproductively isolated from at least one parental species (*G. fortis*) due to differences in song and beak morphology. Whether it is also reproductively isolated from the other parental species (*G. conistrostris*), which resides on the island of Española, remains to be tested. This case shows that reproductive isolation can develop in few generations (but see Hill & Zink, 2018).

## 3 WHAT DOES THE EVIDENCE SAY?

**Figure 1:**
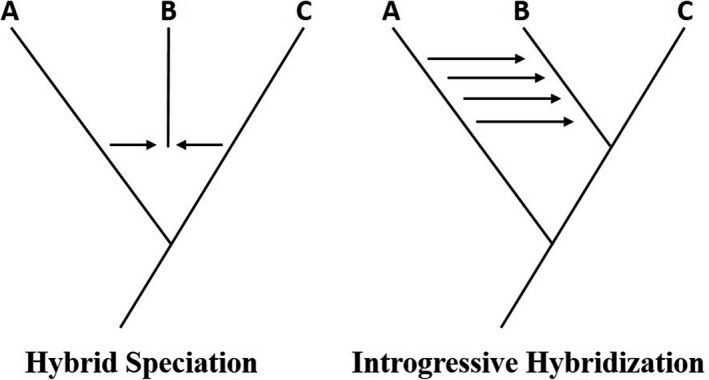
Different evolutionary scenarios can produce genome‐wide signatures of hybridization: hybrid speciation and recurrent introgressive hybridization. Modeling approaches can be applied to discriminate between these two scenarios

This overview of putative hybrid bird species shows that most studies provide convincing genetic and morphological evidence for hybridization (Table 1). However, not all studies could confidently discriminate between hybrid speciation and (recurrent) introgressive hybridization (Figure 1). A complex reticulated evolutionary history involving several bouts of secondary contact can make it difficult—if not impossible—to discriminate between these two scenarios (Mallet, Besansky, & Hahn, 2016). In the case of the golden‐crowned manakin, for example, coalescent modeling showed that speciation models with gene flow were better supported compared to models without gene flow. However, the authors were unable to differentiate between more complex speciation models with gene flow (Barrera‐Guzman et al., 2018a). Phylogenetic network analyses or modeling approaches, such as Approximate Bayesian Computation (ABC), promise to be a fruitful avenue to tackle these issues (Ottenburghs, Kraus, et al., 2017).

**Table 1:** Evidence for the putative hybrid bird species based on three criteria: (a) genetic or morphological evidence for hybridization, (b) reproductive isolation from parental taxa, and (c) reproductive isolation due to hybridization. If criterion three is fulfilled, the taxon is a type I hybrid species, if not it is a type II hybrid species

Hybrid species	Parental species	Evidence for hybridization	Reproductive isolation		Reproductive isolation due to hybridization	Verdict
			Prezygotic	Postzygotic		
Italian Sparrow (*Passer italiae*)	House Sparrow (*P. domesticus*)	Yes	Yes	Yes	No	Type II
	Spanish Sparrow (*P. hispaniolensis*)					
Audubon's Warbler (*Setophaga auduboni*)	Myrtle Warbler (*S. coronata*)	Yes	Yes	Yes	No	Type II
	Black‐fronted Warbler (*S. nigrifrons*)					
Hawaiian Duck (*Anas wyvilliana*)	Mallard (*A. platyrhynchos*)	Yes	Yes	No	?	?
	Laysan Duck (*A. laysanensis*)					
Red‐breasted Goose (*Branta ruficollis*)	Brent Goose (*B. bernicla*)	Yes	Yes	Yes	?	?
	Ancestor of white‐cheeked geese					
Genovesa Mockingbird (*Mimus parvulus bauri*)	San Cristobal Mockingbird (*M. melanotis*)	Yes	?	?	?	?
	Galapagos Mockingbird (*M. parvulus*)					
Golden‐crowned Manakin (*Lepidopthrix vilasboasi*)	Opal‐crowned Manakin (*L. iris*)	Yes	Yes	?	No	Type II
	Snow‐capped Manakin (*L. nattereri*)					
“Bird Bird” (*Geospiza* spp.)	Medium Ground Finch (*G. fortis*)	Yes	Yes	?	Yes	Type I
	Common Cactus Finch (*G. scandens*)					

Note

For three species (Hawaiian duck, red‐breasted goose and Genovesa mockingbird), the evidence is as yet inconclusive.

All putative hybrid species have been documented in only two bird orders: songbirds (Passeriformes) and waterfowl (Anseriformes). Given the incidence of hybridization in birds (Ottenburghs et al., 2015), more hybrid species can be expected. The project to sequence the genomes of all living bird species is currently under way (Jarvis, 2016; Lewin et al., 2018) and will provide abundant resources to explore the evolutionary history of all bird taxa. This endeavor might uncover numerous other hybrid bird species.

## 4 REPRODUCTIVE ISOLATION

Reproductive isolation is mostly caused by the combination of several pre‐ and postzygotic isolation mechanisms. The interplay of these different isolation mechanisms can be depicted as a continuum from a panmictic population to two irreversibly isolated species (Seehausen et al., 2014). In birds, speciation is can be driven by divergent sexual or ecological selection, where prezygotic and extrinsic postzygotic mechanisms act first and intrinsic postzygotic mechanisms come into play later in the speciation process (Price, 2008). The development of reproductive isolation in the putative hybrid bird species can also be visualized on this speciation continuum.

**Figure 2:**
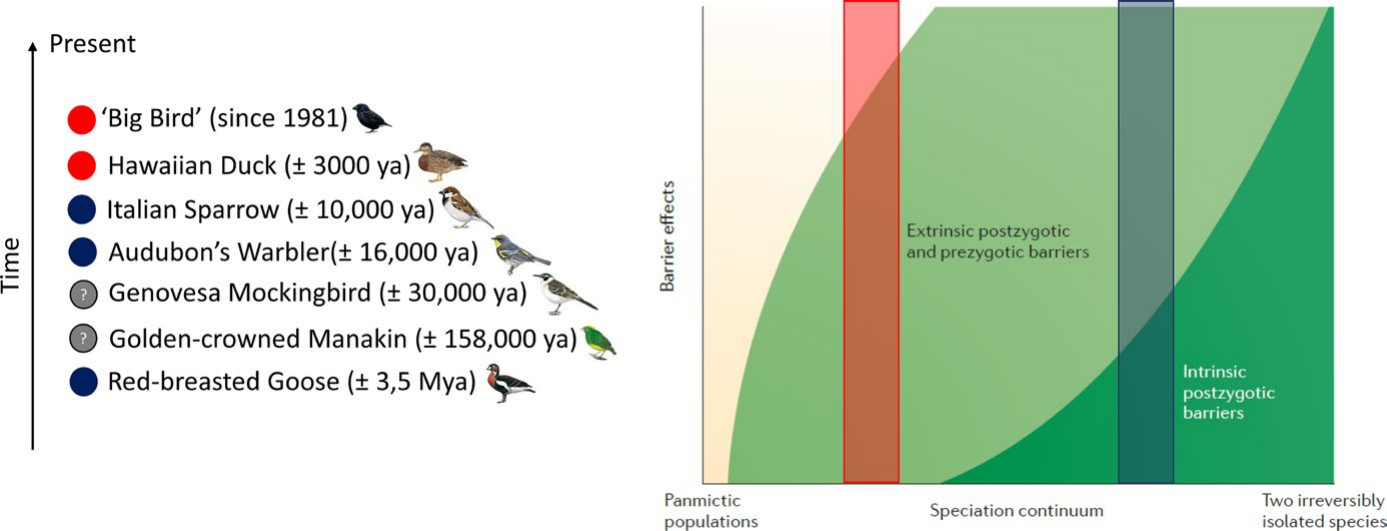
Seven putative hybrid species have originated at different times. The degree of reproductive isolation can be visualized on the speciation continuum from a panmictic population to two irreversibly isolated species (adapted from Seehausen et al., 2014). The hybrid species fall into two broad categories: reproductive isolation by premating mechanisms (red box) and reproductive isolation by intrinsic postzygotic mechanisms (blue box). The curves on the speciation continuum are hypothetical

Based on the available evidence, the location on the speciation continuum of five out of seven hybrid bird species can be inferred (Figure 2). Two species require further research: not much is known about reproductive isolation of the Genovesa mockingbird and the degree of intrinsic postzygotic isolation in the golden‐crowned manakin remains to be determined. In two species (Hawaiian duck and “Big Bird”), reproductive isolation seems to be caused by premating mechanisms, while Italian Sparrow and Audubon's Warbler exhibit some intrinsic postzygotic isolation from their parental species (Brelsford et al., 2011; Trier et al., 2014). Given its age of 3.5 million years, the same probably holds true for the red‐breasted goose, but the nature of intrinsic postzygotic isolation remains to be characterized.

In defining a hybrid species, criterion three stated that hybridization should be the direct cause of reproductive isolation (Schumer et al., 2014). Because this criterion is considered too stringent by some authors (Feliner et al., 2017), I above proposed to discriminate between two types of hybrid species, based on the observation whether reproductive isolation is directly caused by hybridization (type I) or not (type II). Three hybrid speciation events probably involved a period of geographical isolation, resulting in type II hybrid species (i.e., Italian sparrow, Audubon's warbler, and golden‐crowned manakin). Here, hybridization is not the direct cause of reproductive isolation, but some hybrid phenotypes might reinforce it at a later stage. Only “Big Bird” seems to fit the definition of a type I hybrid species where reproductive isolation is directly due to hybridization. In this case, hybridization has resulted in a transgressive bill morphology that separates this hybrid species ecologically and reproductively (through differences in song) from its parental species (Lamichhaney et al., 2018). These examples show that the classification scheme based on criterion three circumvents the epistemological discussion on what constitutes a hybrid species and provides a starting point for further research into the evolution of reproductive isolation. This scheme can be applied to other taxonomic groups (Lavrenchenko, 2014; Mallet, 2007; Mavarez & Linares, 2008; Schumer et al., 2014).

## 5 EVOLUTION OF HYBRID GENOMES

The overview of hybrid bird species revealed hybrid lineages of different ages, ranging from a few generations (“Bird Bird”) over thousands of years (e.g., Italian sparrow and golden‐crowned manakin) to millions of years old (red‐breasted goose). This spectrum of divergence times allows for the comparison of hybrid genome stabilization and adaptation over time, while taking into account species‐specific processes. In the initial stages of first‐generation hybrids and backcrosses, there are many possible outcomes (Nolte & Tautz, 2010). Hybrids might display lower fitness due to disruption of co‐adapted gene complexes or increased fitness due to heterosis (Bar‐Zvi, Lupo, Levy, & Barkai, 2017). In addition, transposable elements might be activated because of mismatches in epigenetic control mechanisms (Fontdevila, 2005; Kapusta & Suh, 2017). On the one hand, these transposable elements can result in deleterious effects such as transcriptomic shock and genome instability (Dion‐Côté, Renaut, Normandeau, & Bernatchez, 2014). On the other hand, they can create novel genetic variation (Belyayev, 2014; Stapley, Santure, & Dennis, 2015). Another issue that hybrid genomes might have to overcome is “hybridization load” where introgression from a species with a smaller effective population size leads to increased genetic load in the hybrids by introducing deleterious alleles. Recent work on swordtail fish (*Xiphophorus*) hybrids showed that alleles from the minor parent are more common in regions of high recombination where they can become uncoupled from deleterious alleles (Schumer, Xu, et al., 2018). We are only starting to understand how hybrid genomes deal with fitness loss/gain, transposable elements, and hybridization load, and how they stabilize over time.

Introgressive hybridization can introduce new genetic variation or create novel allelic combinations (Hedrick, 2013). The mosaic genomes of hybrid species could be a source for adaptive evolution, enabling individuals to exploit habitats that are unavailable for their parental taxa (Dittrich‐Reed & Fitzpatrick, 2013; Roy, Lucek, Walter, & Seehausen, 2015). For example, the transgressive beak morphology of the “Big Bird”‐lineage on the Galapagos islands probably enabled these birds to forage on food resources that are unavailable for other finch species (Lamichhaney et al., 2018). Similar patterns of hybrid ancestry and local adaption of beak morphology have been described for island populations of the Italian sparrow (Bailey, Eroukhmanoff, & Sætre, 2013; Runemark, Fernández, Eroukhmanoff, & Sætre, 2018) and has been suggested for geese (Ottenburghs, Megens, et al., 2017). The interplay between stabilization of hybrid genomes and adaptation to local ecological conditions is a promising field for further research.

## AUTHOR CONTRIBUTIONS

JO conceived the idea, conducted the literature search and wrote the manuscript.

## DATA ACCESSIBILITY

The manuscript is solely based on a literature review. All data can be found in the reference list below.
